# Acute Kidney Injury in Patients After Cardiac Arrest: Effects of Targeted Temperature Management

**DOI:** 10.3390/life15020265

**Published:** 2025-02-10

**Authors:** Silvia De Rosa, Sergio Lassola, Federico Visconti, Massimo De Cal, Lucia Cattin, Veronica Rizzello, Antonella Lampariello, Marina Zannato, Vinicio Danzi, Stefano Marcante

**Affiliations:** 1 Department of Anesthesiology and Intensive Care, San Bortolo Hospital, 36100 Vicenza, Italylucia.cattin@aulss8.veneto.it (L.C.); veronica.rizzello@aulss8.veneto.it (V.R.); antonella.lampariello@aulss8.veneto.it (A.L.); marina.zannato@aulss8.veneto.it (M.Z.); vinicio.danzi@aulss8.veneto.it (V.D.); stefano.marcante@aulss8.veneto.it (S.M.); 2 Centre for Medical Sciences—CISMed, University of Trento, Via S. Maria Maddalena 1, 38122 Trento, Italy; 3 Anesthesia and Intensive Care, Santa Chiara Regional Hospital, APSS, 38121 Trento, Italy; 4 Anaesthesia and Intensive Care, Padova University Hospital, 35128 Padua, Italy; 5 International Renal Research Institute of Vicenza, (IRRIV Foundation), Department of Nephrology, Dialysis and Kidney Transplantation, San Bortolo Hospital, 36100 Vicenza, Italy; massimo.decal@aulss8.veneto.it

**Keywords:** cardiac arrest, acute kidney injury, ischemia–reperfusion injury, targeted temperature management, renal outcomes, critical care

## Abstract

Background: Cardiac arrest (CA) is a leading cause of mortality and morbidity, with survivors often developing post-cardiac arrest syndrome (PCAS), characterized by systemic inflammation, ischemia–reperfusion injury (IRI), and multiorgan dysfunction. Acute kidney injury (AKI), a frequent complication, is associated with increased mortality and prolonged intensive care unit (ICU) stays. This study evaluates AKI incidence and progression in cardiac arrest patients managed with different temperature protocols and explores urinary biomarkers’ predictive value for AKI risk. Methods: A prospective, single-center observational study was conducted, including patients with Return of Spontaneous Circulation (ROSC) post-cardiac arrest. Patients were stratified into three groups: therapeutic hypothermia (TH) at 33 °C, Targeted Temperature Management (TTM) at 35 °C, and no temperature management (No TTM). AKI was defined using KDIGO criteria, with serum creatinine and urinary biomarkers (TIMP-2 and IGFBP7) measured at regular intervals during ICU stay. Results: AKI incidence at 72 h was 31%, varying across protocols. It was higher in the No TTM group at 24 h and in the TH and TTM groups during rewarming. Persistent serum creatinine elevation and fluid imbalance were notable in the TH group. Biomarkers indicated moderate tubular stress in the TTM and No TTM groups. Conclusions: AKI is a frequent complication post-cardiac arrest, with the rewarming phase identified as critical for renal vulnerability. Tailored renal monitoring, biomarker-guided risk assessment, and precise temperature protocols are essential to improve outcomes.

## 1. Introduction

Cardiac arrest (CA) is a critical condition with a high mortality rate and poor prognosis [1]. According to 2021 data from the European Resuscitation Council, the annual incidence of in-hospital cardiac arrest (IHCA) in Europe ranges from 1.5 to 2.8 per 1000 hospital admissions, with 30-day survival rates between 15% and 34% [2,3]. For out-of-hospital cardiac arrest (OHCA), the incidence is higher, ranging from 67 to 170 per 100,000 inhabitants [4], yet the survival-to-hospital discharge rate averages only 8% [5]. Survivors of cardiac arrest often develop post-cardiac arrest syndrome, a systemic condition resulting from the activation of the innate immune system [6]. This syndrome shares features with sepsis, including intravascular volume depletion, vasodilation, endothelial damage, and microcirculatory dysfunction [6,7]. Beyond ischemic damage caused by cardiac arrest itself, ischemia–reperfusion injury (IRI) during the restoration of spontaneous circulation (ROSC) exacerbates systemic harm, leading to multiorgan dysfunction [8,9]. The kidneys are particularly vulnerable to IRI and the hemodynamic instability that follows cardiac arrest [10]. Acute kidney injury (AKI) is a common complication in post-arrest patients [11,12] and is associated with increased mortality, the need for renal replacement therapy (RRT), and prolonged hospital stays [10,12,13]. AKI arises from a combination of direct renal ischemia–reperfusion injury and systemic inflammation, compounded by renal hypoperfusion caused by post-ROSC shock [10,14]. Temperature management has become a cornerstone of post-arrest care, especially for neuroprotection [15]. Recent guidelines, including American Heart Association (AHA) 2023 [15] and International Liaison Committee on Resuscitation (ILCOR) 2024 [16], recommend maintaining a target core temperature of 32–37.5 °C for at least 24 h in comatose post-arrest patients. While Targeted Temperature Management (TTM) offers benefits such as reduced oxidative stress, suppressed inflammation, and lower metabolic demands, it may also have hemodynamic consequences that adversely affect renal function [17]. Hypothermia induces peripheral vasoconstriction and cold diuresis, followed by systemic hypotension and renal hypoperfusion, potentially increasing the risk of AKI [9]. Despite the established neuroprotective role of TTM [18], its impact on renal outcomes remains unclear. Previous studies have produced conflicting results regarding the ability of TTM to prevent AKI, partly due to variability in study designs and the absence of standardized definitions for AKI. At our institution, initial protocols implemented therapeutic hypothermia (TH) at 33 °C. However, considering evolving guidelines and emerging evidence, the protocol was adjusted to TTM at 35 °C, allowing for a comparative evaluation of these two strategies alongside a group of patients not subjected to temperature management (No TTM). This study aims to evaluate the incidence and progression of AKI in a cohort of successfully resuscitated cardiac arrest patients. Specifically, it investigates the effect of temperature management protocols (TH at 33 °C and TTM at 35 °C) on renal function and outcomes, with a focus on the critical rewarming phase. In addition, the study explores the predictive value of TIMP-2 and IGFBP7, urinary biomarkers associated with cellular stress, in assessing AKI risk. By integrating these objectives, this research seeks to provide novel insights into the renal implications of temperature management strategies in post-cardiac arrest care.

## 2. Materials and Methods

### 2.1. Study Design

This was a prospective, single-center, observational study conducted through a collaboration between the Nephrology and Anesthesia and Intensive Care Departments at San Bortolo Hospital in Vicenza. The study protocol was approved by the Ethics Committee, the approval, as clinical protocol, was given from the Institutional Review Board of San Bortolo Hospital in Vicenza. The requirement to obtain informed consent was waived. This study was performed according to the ethical principles of the Declaration of Helsinki. The study was carried out from January 2013 to May 2019 and divided into two timeframes. Between January 2013 and March 2015, patients with GCS ≤ 8 after ROSC were treated with a TH protocol targeting a temperature of 33 °C. Following changes in post-ROSC management guidelines, an amendment to the study was requested. From March 2016 to May 2019, the target temperature was adjusted to 35 °C (TTM). Patients treated with TH or TTM were compared with successfully resuscitated patients who were not subjected to post-arrest temperature control (No TTM).

### 2.2. Patient Selection Criteria

Patients with ROSC following cardiac arrest were enrolled, regardless of the presenting rhythm (asystole, PEA, ventricular fibrillation, or pulseless ventricular tachycardia) or whether the arrest occurred in-hospital or out-of-hospital. Approximately 85% of patients (90 out of 106) received post-arrest temperature management protocol. The TTM protocol excluded patients with the following:

- A time interval between ROSC and the initiation of cooling exceeding 6 h.
- An initial body temperature <30 °C after ROSC.
- Septic shock, known coagulopathy, or active bleeding.
- Major trauma, recent major surgery (<72 h), or severe burns.

Persistent shock post-ROSC was not considered a contraindication, but close invasive monitoring (e.g., Vigileo, Swan-Ganz, or LiDCO) was recommended.

### 2.3. Clinical Management

All patients were managed according to the 2010 and 2015 European Resuscitation Council guidelines, depending on their enrollment period. ROSC was defined as an organized rhythm with a palpable pulse sustained for at least 20 min. The decision to initiate TH or TTM was left to the discretion of the attending physician. For patients in the TH or TTM groups, cooling was performed according to standardized hospital protocols using either the Arctic Sun^®^ 5000 (Brad medical, Denver, CO, USA) or Blanketrol^®^ III (Cincinnati Sub-Zero, Cincinnati, OH, USA); devices. Temperature management included the following:

- Sedation: Propofol (2 mg/kg bolus—if feasible and safe—then 0.5–3 mg/kg/h infusion) or midazolam (0.03–0.04 mg/kg bolus, then 0.2 mg/kg/h infusion).
- Analgesia: Continuous infusion of remifentanil (0.02–0.2 mcg/kg/min) or fentanyl (1–2 mcg/kg/h).
- Paralysis: Continuous infusion of cisatracurium (2 mcg/kg/min).

Other treatments, such as fluid resuscitation and vasopressor administration, were left to the discretion of the attending physician.

For TH-treated patients, the protocol included four phases.

1. Cooling phase to 33 °C.
2. Maintenance phase at 33 °C for 24 h.
3. Rewarming phase to 36 °C at 0.25–0.5 °C/h.
4. Normothermia phase at 36 °C for 48 h.

The TTM-treated patients followed a similar protocol but with a target temperature of 35 °C, a slower rewarming rate (0.15 °C/h), and a final maintenance temperature of 36.5 °C for 48 h.

### 2.4. Sample Handling and Data Collection

Demographic and pre-hospital variables were prospectively collected at admission using Utstein-style criteria. Hemodynamic variables and temperatures were continuously monitored in the ICU, with serial measurements of serum creatinine (sCr). The Nephrocheck test [19] was performed in some TTM-treated and non-TTM-treated patients. Data collected at admission and at 6, 24, 48, and 72 h included core body temperature, sCr, urinary output (UO), fluid balance (FB), furosemide dose, Mean Arterial Pressure (MAP), and Vasoactive Inotropic Score (VIS). AKI was defined using the 2012 KDIGO criteria, relying exclusively on serum creatinine levels for staging. Urinary output and estimated glomerular filtration rate (eGFR) were excluded from the staging process to ensure consistency in the data analysis and account for potential inaccuracies in urine output measurements in critically ill patients [20]. Staging was determined based on the relative changes in sCr from baseline, measured as follows:

- Stage 1: An increase in sCr of ≥0.3 mg/dL within 48 h or 1.5–1.9 times the baseline value.
- Stage 2: An increase in sCr of 2.0–2.9 times the baseline value.
- Stage 3: An increase in sCr to ≥3.0 times the baseline value, sCr ≥ 4.0 mg/dL, or the initiation of renal replacement therapy.

Baseline creatinine was defined as the pre-admission sCr (up to six months before the cardiac event) or estimated using the MDRD formula [21]. Serum creatinine levels were corrected for fluid balance and recorded at admission and at 6, 24, 48, and 72 h [21]. The Sequential Organ Failure Assessment (SOFA) score was calculated using standard methods. To provide a more comprehensive assessment of renal function and AKI progression, additional variables were analyzed, including urinary output, fluid balance, and the urinary biomarkers TIMP-2 and IGFBP7. These biomarkers were evaluated for their potential to detect early tubular stress and stratify AKI risk.

### 2.5. Fluid Balance-Corrected Serum Creatinine

To account for the effects of fluid administration or loss on renal function markers, serum creatinine levels were corrected based on the net fluid balance recorded at each time point. This correction was performed by dividing the total serum creatinine by the patient’s fluid balance (measured as cumulative input minus output) to mitigate the dilutional or concentration effects associated with fluid shifts. This approach ensures a more accurate representation of renal function and aligns with previous studies focusing on critically ill patients.

### 2.6. Outcomes

The primary outcome was the development of AKI in successfully resuscitated patients from ICU admission to 72 h. Secondary outcomes included changes in sCr, urinary output, fluid balance, and the correlation between Nephrocheck and sCr.

### 2.7. Statistical Analysis

Data distribution was assessed using the Shapiro–Wilk test. Given the non-normal distribution of the data, non-parametric statistical methods were applied. Continuous variables are presented as median and interquartile range (IQR) for the TH (therapeutic hypothermia), TTM (Targeted Temperature Management), and No TTM groups. Group differences were evaluated using the Kruskal–Wallis test, while pairwise comparisons between the TH and TTM groups were performed using the Mann–Whitney U test. Categorical variables are expressed as percentages and were analyzed using the Chi-square test. A *p*-value < 0.05 was considered statistically significant. Correlations between quantitative variables were evaluated using the Spearman correlation coefficient. This statistical approach ensures rigorous evaluation of both continuous and categorical data, accommodating their non-parametric nature and providing robust comparisons across the study groups. Data were analyzed using STATA 12 software (STATA corp, 490, Lakeway Drive College Station, 77845, TX, USA).

## 3. Results

### 3.1. Study Population

Between January 2013 and May 2019, 397 patients with a diagnosis of post-cardiac arrest ROSC (Return of Spontaneous Circulation) were admitted to the Department of Anesthesia and Intensive Care at San Bortolo Hospital in Vicenza.

The population was divided into two groups based on the treatment era.

- ***Historical Control Group*** (January 2013–March 2015): Patients treated with TH at 33 °C, in line with the guidelines effective during this period.
- ***Study Group*** (January 2016–May 2019): Patients eligible for temperature control were treated with TTM at 35 °C following revised ROSC management guidelines.

**Figure 1:**
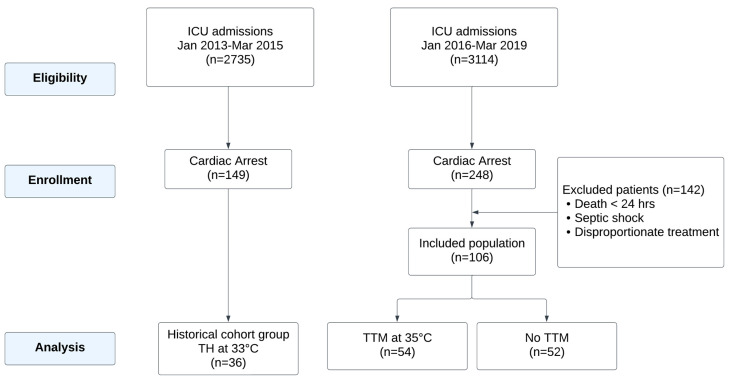
Flowchart of cardiac arrest patient management and inclusion criteria.

Of these, 149 patients were admitted during the 2013–2015 period (historical control group). A total of 113 patients in this group were excluded from the study for the following reasons: death within 24 h of ICU admission, concurrent septic shock, or care deemed disproportionate to their clinical condition. These exclusion criteria were applied to ensure a uniform study population and minimize confounding factors that could affect the analysis of AKI and temperature management outcomes. For the 2016–2019 period (study group), 248 patients were evaluated. Among these, 142 patients were excluded based on similar criteria, resulting in a final cohort of 106 patients. Of these, 54 patients underwent Targeted Temperature Management (TTM) due to a GCS ≤ 8 after ROSC, while the remaining 52 patients, with a GCS > 8, did not receive TTM (Figure 1).

### 3.2. Demographic Data, CPR Quality, and Comorbidities

Table 1 highlights the demographic characteristics, CPR quality, and comorbidities across the three subgroups: TH 33 °C, TTM 35 °C, and No TTM. The cohort (*n* =142) consisted of 71.8% male patients with a median age of 62 years (52–74). No significant differences were found among the subgroups for age, BMI, SOFA score, SAPS II score, the incidence of re-arrest after ROSC, or comorbidities such as hypertension, chronic kidney disease, or COPD. However, the presenting rhythm of cardiac arrest significantly differed among the groups (*p* = 0.008), with shockable rhythms (VF/VT) being more prevalent in the TH 33 °C (83.4%) and TTM 35 °C (68.5%) groups compared to the No TTM group (49.0%). Patients treated with TH 33 °C or TTM 35 °C also received a greater number of shocks and higher doses of adrenaline during resuscitation. Certain comorbidities were also significantly different. Prior cardiac surgery was more common in the TTM 35 °C group, while ischemic heart disease and insulin-dependent diabetes mellitus were more prevalent in the TH 33 °C group (*p* = 0.038 and *p* = 0.041, respectively). As expected, GCS scores after ROSC were significantly higher in the No TTM group, indicating better initial neurological recovery (*p* = 0.010).

### 3.3. Renal and Hemodynamic Outcomes

The renal and hemodynamic outcomes are summarized in Table 2. Baseline sCr levels were significantly higher in the TH 33 °C group (1.06 mg/dL) compared to TTM 35 °C (0.83 mg/dL) and No TTM (0.82 mg/dL, *p* < 0.001). At 72 h, sCr levels remained significantly higher in the TH 33 °C group (1.07 mg/dL) compared to the other groups: TTM 35 °C (0.87 mg/dL) and No TTM (0.81 mg/dL, *p* = 0.037).

**Figure 2:**
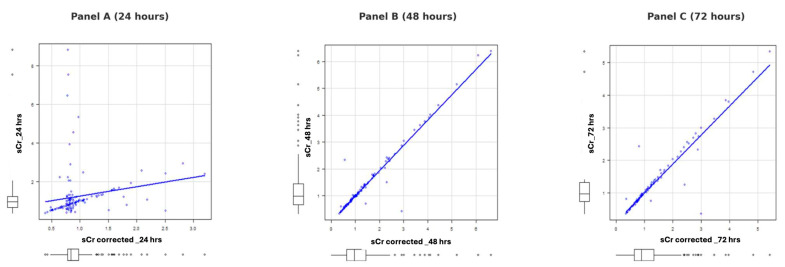
Progression of correlation between sCr and fluid balance-corrected sCr over 24, 48, and 72 h.

Figure 2 illustrates the progression of the correlation between sCr and fluid balance-corrected sCr over 24, 48, and 72 h across the three subgroups. At 24 h (Panel A), the correlation is weak to moderate, with significant dispersion of data points around the regression line, suggesting variability due to fluid balance or other confounding factors. By 48 h (Panel B), the relationship becomes stronger and more linear, indicating improved fluid balance stability. At 72 h (Panel C), the correlation is nearly perfect, demonstrating the reliability of corrected sCr values for clinical monitoring as fluid balance becomes increasingly predictable.

The three images represent the correlation between sCr and fluid balance-corrected sCr at 24, 48, and 72 h, respectively. In Panel A (24 h), the correlation is weak to moderate, with significant dispersion of data points around the regression line, suggesting considerable variability due to fluid balance or other confounding factors. In Panel B (48 h), the relationship becomes stronger and more linear, with data points distributed closer to the regression line and reduced variability, indicating greater stability in fluid balance. Finally, in Panel C (72 h), the correlation is extremely strong, with data points nearly perfectly aligned along the regression line, demonstrating an almost perfect relationship between sCr and corrected sCr. This progression indicates that fluid balance becomes increasingly stable and predictable over time, improving the reliability of corrected sCr values for clinical monitoring.

**Figure 3:**
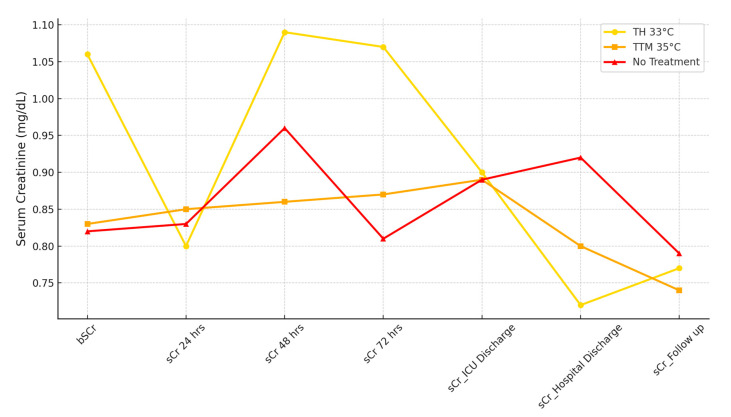
Serum creatinine trends across 24, 48, and 72 h. This figure depicts the progression of sCr levels at admission, 24 h, 48 h and 72 h, ICU discharge, hospital discharge, and follow-up across the three subgroups: TH 33 °C, TTM 35 °C, and No TTM.

Figure 3 illustrates sCr trends over time for the three subgroups. While baseline levels showed significant differences, no significant variation was observed at 24 or 48 h. However, at 72 h, the sCr level in the TH 33 °C group remained elevated compared to the TTM 35 °C and No TTM groups. Similarly, sCr values at hospital discharge were significantly higher in the No TTM group, highlighting differences in renal function outcomes. Urinary output increased from 24 to 72 h across all groups, with significantly higher values at both 24 and 72 h in the TH 33 °C group. Diuretic use did not differ among the groups, and no correlation was observed between urinary output and diuretic therapy. Median fluid balance at 48 h was significantly more positive in the TH 33 °C group (1522 mL) compared to TTM 35 °C (382 mL) and No TTM (228 mL, *p* = 0.003). The mean MAP values over three days remained above 70 mmHg and were similar across groups. While VIS values showed no statistically significant differences, a slight increase was noted in the TTM 35 °C group. Intra-aortic balloon pump (IABP) placement was more frequent in the TH 33 °C group, although not statistically significant.

### 3.4. ICU and Ventilation Outcomes

Early coronary angiography (<6 h) was performed in 43% of the cohort, with no significant differences among the subgroups. The ICU mortality rate was 35.2%, with no significant differences among the groups. However, ICU stay was significantly longer in the TTM 35 °C (10 days) and TH 33 °C (9 days) groups compared to the No TTM group (2 days, *p* < 0.001). Similarly, mechanical ventilation duration was significantly longer in the TTM 35 °C (9.5 days) and TH 33 °C (8 days) groups compared to the No TTM group (2 days, *p* < 0.001).

### 3.5. Development of AKI and Target Temperature Maintenance Times

Based on KDIGO classification, 84.5% of patients (120/142) did not develop AKI at 24 and 48 h, while 15.5% (22/142) presented with AKI at these time points. At 72 h, the incidence of AKI increased to 31% (25/142). Among the three subgroups (TH 33 °C, TTM 35 °C, and No TTM), there was no significant difference in AKI incidence at 72 h (*p* = 0.07). However, differences were significant at earlier time points. At 24 h, AKI was more common in the No TTM group, while at 48 h, it was significantly higher in the TH group (41.7%) and the TTM group (37.1%) compared to the No TTM group (18.8%) (Table 3).

For the Nephrocheck test, which measures the product of urinary biomarkers [TIMP-2] × [IGFBP7], the mean biomarker concentration in the general population was 1.15 (0.38–3.18). Patients treated with TH did not undergo the test. In the TTM group, the median value was 1.54 (0.8–3.46), and in the No TTM group, it was 1.0 (0.19–2.18), with no significant difference between the two groups (*p* = 0.105). Table 3 summarizes AKI incidence by KDIGO stage, Nephrocheck risk classes, and sCr at admission. The analysis of the temperature control subgroups (TH and TTM) revealed significant differences in temperature maintenance and rewarming times. The detailed times for the induction, maintenance, and rewarming phases are provided in Table 4.

## 4. Discussion

### 4.1. Major Findings

This study demonstrates that AKI remains a common complication in post-cardiac arrest patients, with an incidence of 31% at 72 h. AKI was observed in 17.3% of the No TTM group, compared to 13.9% in the TH group and 14.9% in the TTM group at 24 h, highlighting its early prevalence in the No TTM group. Notably, both the TH and TTM groups showed increased AKI incidence during the rewarming phase, indicating that this period represents a critical window of vulnerability. This emphasizes the need for precise temperature monitoring, particularly during rewarming, to mitigate adverse renal outcomes.

### 4.2. Role of Hypothermia in Renal Protection

Hypothermia mitigates post-reperfusion renal injury primarily by modulating the mechanisms of IRI. During IRI, the kidney undergoes oxidative stress, inflammation, and apoptotic cascades [9]. Hypothermia provides protection through multiple pathways. It reduces oxygen consumption and slows enzymatic reaction rates, which helps preserve mitochondrial function and prevent ATP depletion (1). This limits ion dysregulation, intracellular calcium accumulation, and activation of destructive enzymes like proteases and phospholipases [9]. Hypothermia also reduces the production of reactive oxygen species (ROS), preventing lipid peroxidation and mitochondrial dysfunction. Increased endogenous antioxidant activity, such as catalase, during hypothermia further protects against oxidative damage [9].

The anti-inflammatory effects of hypothermia are equally significant. Cooling downregulates pro-inflammatory cytokines, including TNF-α and interleukins, reducing endothelial activation and leukocyte infiltration [9] (16). This helps preserve microvascular integrity and prevents the “no-reflow phenomenon” [9] (18). Furthermore, hypothermia maintains the structural integrity of renal tubular cells, protecting cell membranes, cytoskeletal structures, and tight junctions, which collectively reduce apoptosis and necrosis [9].

### 4.3. Impact of Cold Diuresis and Rewarming on Renal Function

Hypothermia-induced cold diuresis represents a well known phenomenon characterized by increased urine output secondary to peripheral vasoconstriction and central volume redistribution. In our study, the TH group showed significantly higher urine output during the cooling phase compared to the No TTM group. While initially beneficial for maintaining renal perfusion, cold diuresis disrupts water and electrolyte handling, potentially leading to tubular stress. The persistent serum creatinine elevation observed in the TH group suggests impaired tubular reabsorption, despite the increased urine output. This aligns with previous reports that link cold diuresis to significant electrolyte losses, particularly sodium and potassium, which may exacerbate tubular dysfunction if not carefully managed [12,23].

The rewarming phase also plays a critical role in AKI progression. Rapid rewarming was shown to exacerbate oxidative stress, hemodynamic instability, and tubular injury [24]. A slow and controlled rewarming protocol is essential to minimize renal vulnerability during this phase.

### 4.4. Biomarkers in AKI Risk Stratification

Laboratory biomarkers such as TIMP-2 and IGFBP7 have emerged as critical tools in AKI risk stratification. These markers identify tubular stress before serum creatinine or urinary output changes, providing an opportunity for early intervention [22,23]. In our study, Nephrocheck ([TIMP-2] × [IGFBP7]) values exceeded the risk threshold of 0.3, indicating moderate tubular stress across groups. Previous studies have validated TIMP-2 and IGFBP7 as reliable predictors of AKI within three hours of ischemic insult [25]. Additional biomarkers such as NGAL and KIM-1 offer insights into site-specific damage, with NGAL correlating to tubular injury severity and KIM-1 indicating proximal tubular stress [26].

During hypothermia, cold diuresis and fluid shifts can lead to electrolyte imbalances and tubular stress. Despite elevated urine output in the TH group, persistent serum creatinine elevation suggests impaired tubular reabsorption. This pattern aligns with cold-induced diuresis mechanisms disrupting renal water and electrolyte handling [12]. Rapid rewarming during TH and TTM is another critical phase that exacerbates oxidative stress and renal vulnerability, underscoring the importance of slow, controlled rewarming protocols [24].

Ensuring accurate management of T core can reduce tubular stress and reperfusion injury risks. This highlights the importance of employing standardized temperature control protocols and continuous monitoring devices to optimize renal outcomes. Long-term renal outcomes appeared worse in the No TTM group, despite better baseline neurological and hemodynamic profiles.

### 4.5. Acute Kidney Injury After Cardiac Arrest: Integration of Study Findings, Biomarkers, and Literature Comparison

The results of this study provide a comprehensive analysis of AKI incidence and its temporal progression among post-cardiac arrest patients managed with TH, TTM, or No TTM protocols. At 72 h, the incidence of AKI, classified using KDIGO criteria, was 31%, aligning with findings from similar cohorts [12]. Differences in diagnostic criteria, timeframes, and confounding factors likely contribute to these discrepancies. In our study, AKI was more prevalent at 24 h in the No TTM group, reflecting the early impact of ischemia–reperfusion injury and hemodynamic instability. Conversely, both the TH and TTM groups demonstrated a significant increase in AKI incidence during the rewarming phase, a period known to be associated with oxidative stress, altered renal perfusion, and tubular vulnerability. These findings are consistent with De Rosa et al. [24], who reported that rapid rewarming during therapeutic hypothermia could exacerbate tubular injury and worsen renal outcomes. This underscores the rewarming phase as a critical period requiring enhanced vigilance and precise temperature control. Serum creatinine levels remained persistently elevated in the TH group across all time points, despite higher urinary output observed in the same group. This pattern aligns with findings from previous studies and can be attributed to cold-induced diuresis, a well known effect of therapeutic hypothermia [9]. This condition disrupts tubular water and electrolyte handling, resulting in increased hourly urine output and subsequent electrolyte loss through urine [27,28,29,30,31,32]. Although some smaller studies have investigated urine output and electrolyte losses during TTM [33,34], data remain scarce regarding the interaction between serum electrolytes and urine output/excretion in OHCA patients undergoing TTM in the ICU. Furthermore, many existing conclusions are derived from studies with a high risk of bias, underscoring the need for more robust research in this area. In addition, positive fluid balances were most pronounced in the TH group, suggesting a potential mismatch between fluid administration and renal excretion. These results highlight the need for tailored fluid resuscitation strategies and the close monitoring of fluid balance to prevent additional renal stress. While MAP values remained stable across all groups, the complex interplay between hemodynamics, fluid shifts, and renal injury warrants further investigation to optimize fluid and hemodynamic management during TTM protocols [17]. Biomarkers play a critical role in AKI risk stratification and early detection. According to Hou et al. [34], biomarkers such as CRP, LDH, and ALP provide insights into the mechanisms of AKI. Adler et al. [27] demonstrated that urinary biomarkers TIMP-2 and IGFBP7 accurately predict the development of AKI in out-of-hospital cardiac arrest survivors as early as 3 h after the event. With a cut-off value of 0.24, the test showed a sensitivity of 96.8% and a specificity of 94.1% [27]. This approach may facilitate the early identification of high risk patients, improving clinical management and therapeutic strategies. In our study, the Nephrocheck test revealed intermediate biomarker values in both groups, with no statistically significant difference observed (*p* = 0.105). Nevertheless, both groups exceeded the commonly accepted threshold of 0.3, suggesting a moderate risk of tubular stress across the cohort. Godi et al. [19] highlighted that the Nephrocheck test ([TIMP-2] × [IGFBP7]) is a valuable tool for early AKI detection, capable of identifying tubular stress before changes in serum creatinine or urinary output become apparent. However, the test primarily serves as a risk stratification tool rather than a direct diagnostic marker of AKI.

### 4.6. Strengths, Limitations, and Future Directions

This study has several strengths that enhance its contribution to the understanding of AKI in post-cardiac arrest patients. First, it provides a comprehensive temporal analysis of AKI incidence across three temperature management protocols. Second, integrating biomarkers such as Nephrocheck offers a more nuanced understanding of renal tubular stress and injury mechanisms. Finally, identifying the rewarming phase as a critical period for AKI risk underscores the importance of targeted monitoring during this phase.

However, several limitations should be acknowledged to provide a comprehensive understanding of the findings. Being a retrospective, single-center study with a relatively small sample size, the results may not be fully generalizable. The use of the MDRD formula to estimate baseline creatinine may have introduced AKI misclassification. However, TIMP-2 and IGFBP7 were selected due to their established clinical utility and availability at our institution as part of the NephroCheck test, specifically designed for early detection of tubular stress in critically ill patients. The inclusion of additional biomarkers, such as beta-2-microglobulin, KIM-1, or NGAL, which reflect site-specific renal damage, was not feasible due to logistical and resource constraints. Although oxidative stress markers provide valuable insights into post-reperfusion injury, their measurement was beyond the scope of this study due to resource constraints.

Second, while our cohort was carefully selected and stratified, significant baseline differences were present among the groups, including the prevalence of coronary heart disease (CHD) and diabetes. These conditions are known to influence oxidative stress pathways and post-reperfusion injury outcomes. CHD can exacerbate systemic inflammation and endothelial dysfunction, potentially increasing oxidative stress levels, while diabetes, through hyperglycemia-induced oxidative damage and impaired vascular responses, may also amplify these effects. Such differences could have contributed to variability in the oxidative stress markers and renal outcomes observed across the study groups. Although efforts were made to control for confounding variables, the possibility of residual confounding cannot be ruled out.

Furthermore, the rewarming phase, identified as a critical period of renal vulnerability, was managed using standardized protocols. However, individual variations in rewarming rates and hemodynamic responses may have further influenced outcomes. Including more granular data on oxidative stress markers and longer-term follow-up could enhance the understanding of these interactions.

This study incorporated historical data from the TH group, which differs in size and gender composition compared to the other groups. The male predominance in the TH group (80.6%) and No TTM group (78.8%) may have influenced outcomes, as the existing literature indicates potential hormonal effects on ischemia–reperfusion injury and AKI progression. Future research should aim to explore these gender-based differences in greater detail, particularly the protective role of estrogen in renal outcomes. Additionally, the lack of long-term follow-up data prevented an assessment of chronic kidney disease (CKD) outcomes or acute kidney disease (AKD) staging. Lastly, this study was not designed to evaluate the impact of temperature management on the need for renal replacement therapy (RRT).

Looking forward, future research should include the following: (1) Prospective, multicenter studies with larger cohorts and long follow-up periods. (2) The development of standardized temperature management protocols, with emphasis on the rewarming phase. (3) Improved understanding of the sensitivity and specificity of biomarkers such as Nephrocheck. (4) The integration of advanced hemodynamic monitoring tools assess renal perfusion dynamics more precisely. In the absence of tools like renal Doppler or VExUS score, integrating echocardiographic parameters might offer a practical alternative. Measurements such as TAPSE, vena cava plethora, right ventricular function, and left ventricular ejection fraction could provide indirect but meaningful data on renal perfusion and congestion. Including these assessments in clinical protocols could enhance patient management and improve risk stratification, particularly during critical phases like rewarming. (5) The potential confounding role of sepsis, which is a frequent complication in post-cardiac arrest patients. The overlapping mechanisms of systemic inflammation and hypoperfusion seen in sepsis and post-cardiac arrest syndrome make it challenging to disentangle their respective contributions to AKI. Future research should focus on incorporating specific biomarkers or imaging techniques to better differentiate between these conditions and refine AKI management strategies. (6) Long-term studies focusing on chronic renal outcomes and patient quality of life. (7) The gender-based differences in renal outcomes, particularly the potential protective role of estrogen, could yield significant insights into developing tailored therapeutic strategies. (8) The allocation of patients to treatment groups may have introduced biases related to presenting rhythms and initial clinical decisions, which could shape observed outcomes, as discussed above. (9) The oxidative stress parameters in future studies is critical to advancing the understanding of oxidative mechanisms in temperature management and AKI. Additionally, incorporating a broader panel of biomarkers may provide a more comprehensive evaluation of the complex pathophysiological processes involved.

## 5. Conclusions

AKI remains a frequent and significant complication following cardiac arrest, with implications for both short- and long-term outcomes. While no significant differences in AKI incidence at 72 h were observed between the TH, TTM, and No TTM groups, the rewarming phase emerged as a critical period of renal vulnerability.

Nephrocheck demonstrated a moderate risk of tubular stress, underscoring their role in risk stratification. These findings highlight the importance of personalized renal monitoring strategies, careful fluid balance management, and precise rewarming protocols to reduce AKI risk and improve patient outcomes.

## Figures and Tables

**Table 1:** Demographic data, CPR quality, and comorbidities.

Variable	Entire Cohort (n = 142)	TH 33 °C (n = 36)	TTM 35 °C (n = 54)	No Treatment (n = 52)	*p*-Value
Male (%)	102 (71.8%)	29 (80.6%)	32 (59.3%)	41 (78.8%)	0.102
Age (yrs)	62 (52–74)	60 (53–68)	58 (50–72)	66 (51–76)	0.295
BMI (kg/m^2^)	25.2 (23.8–27.7)	25.8 (23.1–28.2)	24.9 (24.0–26.8)	25.2 (23.7–27.7)	0.967
SOFA score	9 (7–11)	9 (8.2–10.3)	9 (7.5–12.0)	8 (7–11)	0.177
SAPS II score	57 (48–66)	54 (50–60)	61 (52–68)	54 (42–71)	0.079
Cardiac arrest rhythm					0.008
-Shockable (VF/VT) (%)	93 (64.5%)	30 (83.4%)	37 (68.5%)	25 (49.0%)	
-Non-shockable (%)	49 (34.8%)	6 (16.7%)	17 (31.5%)	26 (51.0%)	
Re-arrest after ROSC (%)					0.196
-Shockable (%)	19.0	30.6	18.5	11.5	
-Non-shockable (%)	7.7	2.8	9.3	9.6	
-No re-arrest (%)	73.2	66.7	72.2	78.8	
GCS post-ROSC	3 (3–4)	3 (3–3.25)	3 (3–3)	3 (3–7.5)	0.010
Hypertension (%)	46.5	38.9	44.4	53.8	0.357
Previous renal disease (%)	14.1	13.9	9.3	19.2	0.337
Previous cardiac surgery (%)	4.2	5.6	3.7	3.8	0.038
-CABG	17.6	0.0	25.9	21.2	
-Valve Surgery (%)	0.7	0.0	0.0	1.9	
Coronary artery disease (%)	45.1	66.7	44.4	36.5	0.040
Insulin-dependent diabetes (%)	10.6	22.9	3.7	1.9	0.041
COPD (%)	7.0	8.3	1.9	11.5	0.141

Abbreviations: BMI: body mass index; SOFA: Sequential Organ Failure Assessment; SAPS: Simplified Acute Physiology Score; VF: ventricular fibrillation; VT: ventricular tachycardia; ROSC: Return of Spontaneous Circulation; CABG: Coronary Artery Bypass Graft; COPD: Chronic Obstructive Pulmonary Disease.

**Table 2:** Renal and hemodynamic outcomes.

Variable	Entire Cohort (n = 142)	TH 33 °C (n = 36)	TTM 35 °C (n = 54)	No Treatment (n = 52)	*p*-Value
Baseline Creatinine (mg/dL)	0.84 (0.80–1.02)	1.06 (1.02–1.09)	0.83 (0.80–0.86)	0.82 (0.80–0.89)	<0.001
Creatinine at Admission	1.03 (0.85–1.26)	1.15 (0.95–1.21)	1.02 (0.81–1.26)	1.03 (0.81–1.27)	0.523
Creatinine (Corrected for Fluid Balance)					
- 24 h	0.84 (0.78–0.98)	0.80 (0.65–1.07)	0.85 (0.80–1.06)	0.83 (0.80–0.89)	0.333
- 48 h	0.96 (0.64–1.44)	1.09 (0.80–1.40)	0.86 (0.59–1.73)	0.96 (0.61–1.36)	0.516
- 72 h	0.90 (0.64–1.33)	1.07 (0.79–1.41)	0.87 (0.60–1.47)	0.81 (0–1.13)	0.037
Creatinine at ICU Discharge	0.90 (0.61–1.12)	0.90 (0.42–2.32)	0.89 (0.33–1.12)	0.89 (0.6–1.19)	0.12
Creatinine at Hospital Discharge	0.82 (0.60–1.12)	0.72 (0.57–0.91)	0.80 (0.54–1.02)	0.92 (0.70–1.42)	0.017
Creatinine at Follow-Up	0.78 (0.63–1.0)	0.77 (0.66–0.90)	0.74 (0.56–0.91)	0.79 (0.66–1.49)	0.546
Urinary Output (mL)					
- 24 h	1750 (1080–2762)	2242 (1403–2844)	1552 (964–2408)	1600 (1080–2870)	0.07
- 48 h	2800 (2122–3477)	2877 (2362–3552)	2540 (2105–3487)	2465 (1372–3255)	0.236
- 72 h	2560 (1880–3764)	3185 (2538–4060)	2460 (1665–3482)	2001 (960–3200)	0.004
Fluid Balance (mL)					
- 24 h	49.8 (−931–1092)	333 (−434–1280)	347 (−578–1777)	250 (−1067–1054)	0.43
- 48 h	748 (−196.7–1669)	1522 (488–2236)	382 (−397–1553)	228 (−393–1530)	0.003
- 72 h	418 (−691–1393)	621 (−389–1749)	801 (−503–1552)	−176 (−739–671)	0.17
Cumulative Fluid Balance	1342 (−1073–2853)	2441 (437–4043)	500 (541–2781)	1166 (−1705–2122)	0.034
ICU Stay (Days)	8 (4–14)	9 (4.5–12.5)	10 (7–19)	3 (2–7.25)	<0.001
Ventilation Days	7 (3–12)	8 (4–12.7)	9.5 (5.25–17.7)	2 (1–6.25)	<0.001

**Table 3:** AKI development, Nephrocheck risk class, and sCr values at admission.

Parameter	TH 33 °C (n = 36)	TTM 35 °C (n = 54)	No Treatment (n = 52)	*p*-Value
AKI Development (%)				
AKI at 24 h	5 (13.9)	8 (14.9)	9 (17.3)	0.896
AKI at 48 h	15 (41.7)	20 (37.1)	10 (18.8)	0.047
AKI at 72 h	12 (33.3)	15 (27.8)	17 (32.7)	0.809
KDIGO Staging at 24 h				
KDIGO Stage 1 (%)	5 (13.9)	6 (11.1)	7 (13.5)	0.734
KDIGO Stage 2 (%)	0 (0)	2 (3.7)	2 (3.8)	0.472
KDIGO Stage 3 (%)	0 (0)	0 (0)	0 (0)	-
KDIGO Staging at 48 h				
KDIGO Stage 1 (%)	12 (33.3)	9 (16.7)	5 (9.6)	0.054
KDIGO Stage 2 (%)	2 (5.6)	5 (9.3)	0 (0)	0.029
KDIGO Stage 3 (%)	1 (2.8)	6 (11.1)	10 (19.2)	0.016
KDIGO Staging at 72 h				
KDIGO Stage 1 (%)	8 (22.2)	6 (11.1)	7 (13.5)	0.392
KDIGO Stage 2 (%)	3 (8.3)	4 (7.4)	0 (0)	0.104
KDIGO Stage 3 (%)	1 (2.8)	5 (9.3)	10 (19.2)	0.024
Nephrocheck Results				
Risk Class	Not performed	Intermediate	AKI Subclinical [22]	-
Median Value	Not performed	1.54 [0.8–3.46]	1.0 [0.19–2.18]	0.105
sCr at Admission (mg/dL)	1.15 [0.95–1.21]	1.02 [0.81–1.26]	1.03 [0.81–1.27]	0.742

Abbreviations: AKI: Acute Kidney Injury; sCr: serum creatinine; TH: therapeutic hypothermia; TTM: Targeted Temperature Management; Nephrocheck: AKI biomarker test (IGFBP7 and TIMP-2); IGFBP7: Insulin-like Growth Factor-Binding Protein 7; TIMP-2: Tissue Inhibitor of Metalloproteinases 2.

**Table 4:** Temperature induction, maintenance, and rewarming times.

Time Variable	Entire Cohort	TH 33 °C	TTM 35 °C	*p*-Value
Time to 33 °C (CA onset)	273 [209–390]	280.5 [240–385]	275 [180–395]	0.740
Time to 33 °C (TH Induction)	180 [90–260]	184.5 [120–248]	120 [34–293]	0.329
Maintenance Duration	1440 [1440–1440]	1440 [1440–1557]	1440 [1440–1440]	0.027
Rewarming Time to 36 °C	720 [540–1960]	600 [480–720]	1960 [1041–2880]	<0.001

Abbreviations: CA: cardiac arrest; TH: therapeutic hypothermia; TTM: Targeted Temperature Management.

## Data Availability

The data supporting the reported results are not publicly available due to privacy and ethical restrictions. However, data can be made available upon reasonable request to the corresponding author and with the approval of the Institutional Review Board of San Bortolo Hospital in Vicenza.

## Abbreviations

The following abbreviations are used in this manuscript:

AHA	American Heart Association
AKI	Acute Kidney Injury
CA	cardiac arrest
CKD	chronic kidney disease
COPD	Chronic Obstructive Pulmonary Disease
CRRT	Continuous Renal Replacement Therapy
ICU	Intensive Care Unit
ILCOR	International Liaison Committee on Resuscitation
KDIGO	Kidney Disease: Improving Global Outcomes
MAP	Mean Arterial Pressure
MDRD	Modification of Diet in Renal Disease
No TTM	No Targeted Temperature Management
ROSC	Return of Spontaneous Circulation
sCr	serum creatinine
SOFA	Sequential Organ Failure Assessment
TTM	Targeted Temperature Management
TH	therapeutic hypothermia
TIMP-2	Tissue Inhibitor of Metalloproteinases 2
IGFBP7	Insulin-like Growth Factor-Binding Protein 7
VIS	Vasoactive Inotropic Score
